# Simultaneous spatiotemporal mapping of *in situ* pH and bacterial activity within an intact 3D microcolony structure

**DOI:** 10.1038/srep32841

**Published:** 2016-09-08

**Authors:** Geelsu Hwang, Yuan Liu, Dongyeop Kim, Victor Sun, Alejandro Aviles-Reyes, Jessica K. Kajfasz, Jose A. Lemos, Hyun Koo

**Affiliations:** 1 Biofilm Research Labs, Levy Center for Oral Health, Department of Orthodontics and Divisions of Pediatric Dentistry & Community Oral Health, School of Dental Medicine, University of Pennsylvania, Philadelphia, PA, USA; 2 Department of Oral Biology, University of Florida College of Dentistry, Gainesville, FL, USA

## Abstract

Biofilms are comprised of bacterial-clusters (microcolonies) enmeshed in an extracellular matrix. *Streptococcus mutans* can produce exopolysaccharides (EPS)-matrix and assemble microcolonies with acidic microenvironments that can cause tooth-decay despite the surrounding neutral-pH found in oral cavity. How the matrix influences the pH and bacterial activity locally remains unclear. Here, we simultaneously analyzed *in situ* pH and gene expression within intact biofilms and measured the impact of damage to the surrounding EPS-matrix. The spatiotemporal changes of these properties were characterized at a single-microcolony level following incubation in neutral-pH buffer. The middle and bottom-regions as well as inner-section within the microcolony 3D structure were resistant to neutralization (vs. upper and peripheral-region), forming an acidic core. Concomitantly, we used a green fluorescent protein (GFP) reporter to monitor expression of the pH-responsive *atpB* (P_*atpB*_::*gfp*) by *S. mutans* within microcolonies. The *atpB* expression was induced in the acidic core, but sharply decreased at peripheral/upper microcolony regions, congruent with local pH microenvironment. Enzymatic digestion of the surrounding matrix resulted in nearly complete neutralization of microcolony interior and down-regulation of *atpB*. Altogether, our data reveal that biofilm matrix facilitates formation of an acidic core within microcolonies which in turn activates *S. mutans* acid-stress response, mediating both the local environment and bacterial activity *in situ*.

Biofilms are comprised of dense, highly hydrated clusters of microbial cells that are embedded with and surrounded by an extracellular matrix of polymeric substances, such as exopolysaccharides (EPS), proteins, and nucleic acids^1^,^2^. The production of extracellular matrix by microorganisms enhances cell adhesion and cohesion that promote both microbial accumulation onto a surface and the development of densely packed cell aggregates known as microcolonies^1^,^2^,^3^,^4^. The matrix provides a scaffold for cellular organization into a 3D microenvironment, where cells interact with dynamically changing chemical and physical signals at microscale that can influence bacterial survival and physiological activity within microcolonies^4^,^5^,^6^,^7^. The matrix also provides mechanical stability to biofilms^2^,^8^,^9^, contributing to persistent infections in the human body^1^,^2^. However, the role of the matrix in the microenvironmental heterogeneity and in bacterial activity within microcolonies remains poorly understood^4^,^9^,^10^.

*Streptococcus mutans* is a prototypical biofilm-forming organism and a key oral pathogen associated with dental caries (tooth-decay), one of the most prevalent and costly oral infectious diseases worldwide. In addition to being a highly acidogenic and aciduric organism, *S. mutans* secretes several EPS-producing exoenzymes termed glucosyltransferases that make this bacterium a chief matrix producer in the oral cavity^11^. The EPS promote microbial binding onto the tooth surface and to each other forming highly adherent and cohesive microcolonies^12^,^13^,^14^,^15^. Microscopic images of human dental plaque-biofilm collected from caries-active children also reveal microbial clusters surrounded by EPS^3^. The metabolic activity of bacterial cells within EPS-enmeshed microcolonies help to create localized acidic microenvironments despite the presence of buffering saliva in the oral cavity^13^,^16^. The persistence of acidic microenvironments promotes survival of acidogenic and acid-tolerant microbiota^17^,^18^,^19^, resulting in demineralization of tooth enamel and the onset of dental caries^20^.

Although the importance of the matrix in microcolony development and spatial/chemical heterogeneity has been recognized^21^, studying the local microenvironment and microbial activity within the biofilm without disturbing its natural structure is challenging. Previous *in situ* gene expression studies using fluorescent protein reporters with non-invasive imaging technique such as confocal microscopy has advanced the ability to detect localized gene expression^22^,^23^,^24^,^25^. Furthermore, emerging technologies such as microfluidic and 3D printing approaches have provided additional opportunities to create microenvironments to mimic the spatial confinement and chemical heterogeneity of microcolonies and study their impact on microbial behavior^5^,^7^,^26^,^27^,^28^,^29^,^30^,^31^. However, these artificial cell aggregates based on modelling of confined structures may not reflect the exact properties of native biofilm microcolonies. Recent studies combining high-resolution microscopy and genetic techniques have facilitated microanatomical and spatial order of physiological differentiation and EPS deposition during biofilm formation^32^, which can help to elucidate the influence that bacterial activity and the matrix have on the local microenvironment.

Inspired by these studies, we have developed a novel approach for simultaneous spatio-temporal analysis of pH microenvironments, EPS matrix and gene expression within an intact biofilm at the level of a single microcolony. To investigate directly how microbial cells respond to the surrounding milieu, we used *S. mutans* to assemble EPS-enmeshed microcolonies harboring highly acidic microenvironments. We selected a pH-responsive gene, *atpB* (coding for the beta sub-unit of the F_1_F_0_-ATPase enzyme), that is differentially expressed by *S. mutans* under acidic pH (5.0) versus neutral pH (7.0)^17^,^33^,^34^. Using micro-scale maps of *in situ* pH and gene expression via an *atpB*::GFP reporter, we demonstrated that changes in the pH microenvironment modulate *atpB* activity throughout the 3D structure following incubation in neutral pH buffer. Our data reveal a dome shaped acidic core within the microcolony that is highly resistant to neutralization by the buffering solution. In turn, the local acidity activates the *atpB* expression by *S. mutans* cells residing in the same region. Topical digestion of the surrounding EPS-matrix resulted in nearly complete neutralization of the microcolony interior, and the bacterial *atpB* activity was down-regulated concomitantly. This study provides new insights into how the matrix can modulate both the local pH microenvironment and bacterial activity *in situ* within the microcolony 3D structure.

## Results

### Spatio-temporal analysis of *in situ* pH throughout the intact 3D microcolony

**Figure 1:**
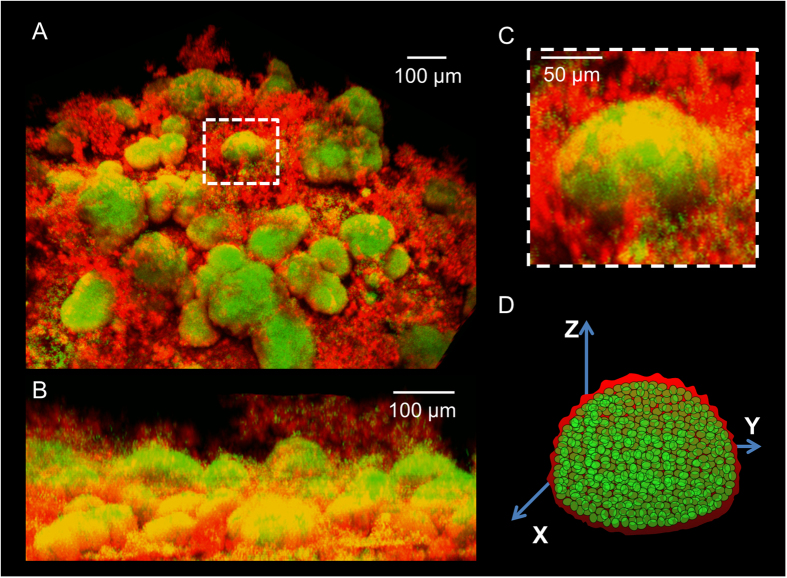
Tri-dimensional (3D) architecture of *Streptococcus mutans* biofilm. (**A**) A representative image of *S. mutans* biofilm comprised of bacterial cell-clusters or microcolonies (green) enmeshed in EPS (red). (**B**) Orthogonal view of the biofilm. (**C**) Magnified single microcolony structure (depicted in white dashed-line box). (**D**) 3D schematic diagram of a single microcolony containing densely-packed bacterial cells.

*S. mutans* cells form well-defined 3D microcolonies while producing an exopolysaccharides (EPS)-rich matrix^12^,^13^. A representative *S. mutans* biofilm 3D architecture (Fig. 1A) and orthogonal images (Fig. 1B) show the microcolony structure (box with white dashed line; Fig. 1A,C) comprised of bacterial cell-clusters (in green; Fig. 1C) enmeshed with EPS (in red; Fig. 1C). 3D schematic diagram of single microcolony containing bacterial cells (in green) is depicted in Fig. 1D. Here, we focused on the microcolonies with a circular-like shape.

**Figure 2:**
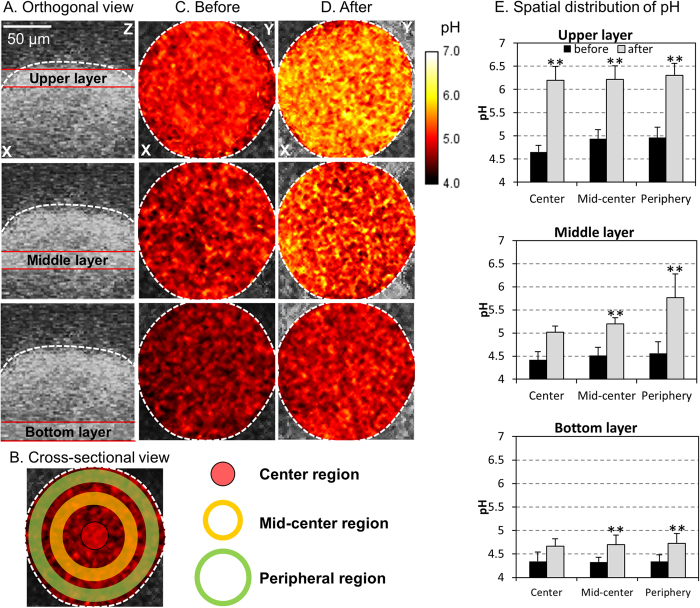
Spatial pH distribution within an intact microcolony. (**A**) Orthogonal and (**B**) cross-sectional sections within the microcolony, which were divided as follows: upper-, middle- and bottom-layer as well as center-, mid-center- and peripheral-region. (**C**) pH distribution at each orthogonal sections before and (**D**) after incubation in neutral pH buffer. (**E**) pH values at each layer and each section of the microcolony. This figure indicates persistence of acidic pH between the bottom and lower-middle layers of the microcolony despite exposure to neutral pH buffer. Double asterisk indicates that the values are significantly different from each other (*P* < 0.01).

**Figure 3:**
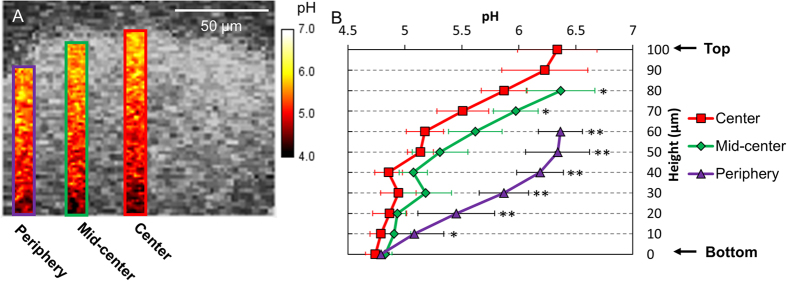
Orthogonal pH distribution within the microcolony. (**A**) Representative images of orthogonal distribution of pH at each region. (**B**) Spatial pH profiles (top-to-bottom) at each region. This figure indicates that the periphery of the microcolony structure can be more effectively neutralized by the pH 7.0 buffer (vs center or mid-center region). Asterisk (*P* < 0.05) and double asterisk (*P* < 0.01) indicate that the values for the different experimental groups are significantly different from each other.

**Figure 4:**
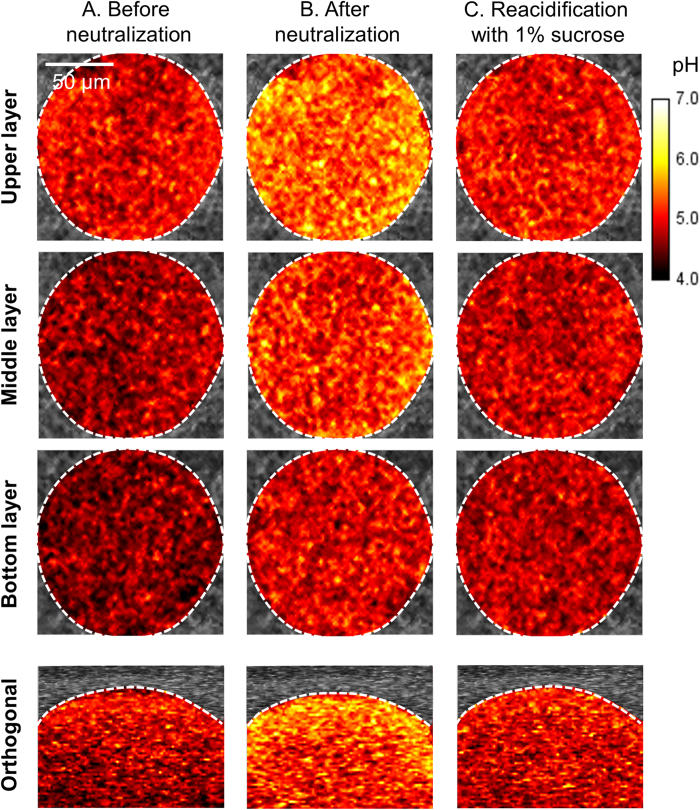
Representative images of cross-sectional and orthogonal pH distribution within the microcolony. (**A**) Before and (**B**) after neutralization, and (**C**) after reacidification of EPS-microcolony. When the biofilms were exposed to sucrose, acidic microenvironments within the microcolony were almost completely reinstated, showing pH distributions similar to those prior to neutralization.

The presence of highly acidic pH values within biofilms despite of the neutral-pH environment found in the oral cavity serves as a major virulence attribute for development of dental caries^35^,^36^. Thus, we analyzed in detail the spatial pH distribution throughout the 3D microcolony structure following incubation in pH 7 buffer (for 60 min). To achieve this, we utilized our pH mapping technique using pH-responsive fluorophore and multi-photon confocal fluorescence microscopy^13^. Although only one representative image is presented, these analyses were performed in quadruplicate and at least 10 images were recorded under confocal microscopy (Figs 2, 3, 4). We initially ‘trisected’ the microcolony orthogonally as upper/middle/bottom layers and cross-sectionally as center/mid-center/peripheral regions (Fig. 2A,B). Then, we profiled the average pH values distributed across the height of intact microcolony before (Fig. 2C) and after neutralization (Fig. 2D). Before neutralization, the majority of microcolony interior exhibited an acidic microenvironment (pH 4 to 5) across the thickness (height) of the microcolony (Fig. 2C and black columns in 2E). Following incubation in neutral pH buffer, we observed that the upper layer of the microcolony was mostly neutralized from center to peripheral regions (Fig. 2D and gray columns in 2E). In contrast, the bottom layer of microcolony (close to the surface of attachment) remained mostly acidic regardless of cross-sectional regions (center or periphery) even after 60 min of incubation in neutral pH buffer. The middle layer was partially neutralized, particularly at the peripheral region (Fig. 2D,E). These observations indicated persistence of acidic pH values from the bottom to lower-middle layers of the microcolony despite exposure to pH 7 buffer.

Furthermore, we also determined the orthogonal (top-to-bottom) pH distribution at the center, mid-center and periphery of the microcolony (Fig. 3). At the center region, low pH (~4.7) was maintained up to 40 μm, while pH values steadily increased as the height increased above 50 μm (Fig. 3B). At mid-center region, the pH profile showed a similar pattern to that of the center region, but displayed greater pH values at each height >50 μm (compared to the center region), indicating increased resistance to neutralization in areas near the bottom (up to 40 μm) and closer to the center of the microcolony (Fig. 3A,B). Notably, in the peripheral region, pH gradually, but constantly, increased over the height (Fig. 3B), indicating that the periphery of the microcolony structure can be more effectively neutralized by the pH 7.0 buffer (vs center or mid-center region).

### Reacidification of microcolony environment by sucrose

**Figure 5:**
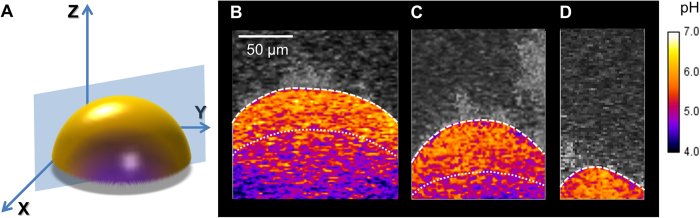
Presence of an inner acidic core within the microcolony . (**A**) Schematic diagram depicting the acidic core in the interior of the microcolony 3D structure. Representative orthogonal images of microcolony with varying sizes: (**B**) 100, (**C**) 60 and (**D**) 30 μm thicknesses. “Fire” lookup table (LUT) color scheme was used for enhanced contrast to differentiate pH ranges. Red-to-yellow colors indicate the area with pH >5 and dark purple-to-pink colors indicate the area with pH <5. The acidic region (under dotted lines) is clearly visible within large microcolonies (100 μm height), while a relatively smaller acidic core was observed in a microcolony with 60 μm height, and it was not detected in microcolonies with less than 30 μm thickness.

Previous studies have suggested that the EPS-matrix can act as a diffusion barrier that limits penetration of charged ions in and out of cariogenic biofilms. However, uncharged solutes such as sucrose may readily diffuse into the biofilm and be utilized for acid production by the embedded bacteria^37^,^38^. To verify this process and reacidification of the interior of the microcolony, we first incubated the biofilm in pH 7 buffer for neutralization (as shown in Figs 2 and 3), followed by addition of 1% (v/v) sucrose, and further incubation for 60 min. Cross-sectional and orthogonal views of microcolony are summarized in Fig. 4. When the biofilms were exposed to sucrose, acidic microenvironments within the microcolony were almost completely reinstated, showing pH distribution profile similar to that prior to neutralization (Fig. 4A,C). Altogether, the three-dimensional pH mapping analyses reveal a compartmentalized area near the bottom layer at the center region of the microcolony that is highly acidic and resistant to neutralization. Based on cross-sectional and orthogonal analyses of spatial pH distribution within the microcolony, we depicted a schematic diagram of the acidic region (in purple; Fig. 5A).

At the same time, it is conceivable that resistance to neutralization and formation of acidic inner region or ‘core’ may be also related to the size of the microcolony. Thus, we visualized several microcolonies of different height (thickness) using “fire” lookup table (LUT) color scheme for enhanced contrast^39^. As expected, the acidic region (as indicated by purple areas below dotted lines) is clearly visible within large microcolonies (100 μm height), which appear dome-shaped based on image rendering (Fig. 5B). Interestingly, a relatively smaller acidic core was observed in a microcolony with 60 μm height (Fig. 5C), while it was not detected in microcolonies with less than 30 μm thickness (Fig. 5D), suggesting that the presence of localized inner acidic region is dependent on microcolony size.

### Mathematical modelling of diffusion

Diffusion (random molecular motion) is an important driving force for transporting solutes into a bacterial cluster (microcolony)^40^. To estimate how fast the solutes from the buffer (citric acid-Na_2_HPO_4_) reach to the core of the microcolony, we employed a simple mathematical diffusion modelling (see Supplementary Information for details), which assumed a microcolony-shaped structure similar to that observed in our biofilm (Fig. 1). The model predicts that neutral buffer solutes could access the interior of large microcolonies (100 μm thickness) in about 10 minutes, while it takes about 3–4 minutes to reach the interior of a medium size microcolony (60 μm), and less than a minute for small size microcolony (30 μm). Because equilibration time scales as the square of the diffusion distance^40^ and neutral pH solution reacts with acids in the vicinity during diffusion, especially at the center and bottom of the microcolony (more acidic vs. outer layers), longer time would be required to neutralize the interior of larger microcolonies (100 μm). These findings are consistent with our experimental results where acidic core was more evident in larger microcolonies. Since the EPS-rich matrix could retard access of neutralizing buffer and help confine acids within the microcolony structure, we hypothesized that treatment with EPS-degrading dextranase would compromise the ability of the matrix to limit diffusion into the microcolony.

### *in situ* pH profile of a microcolony with matrix damage

**Figure 6:**
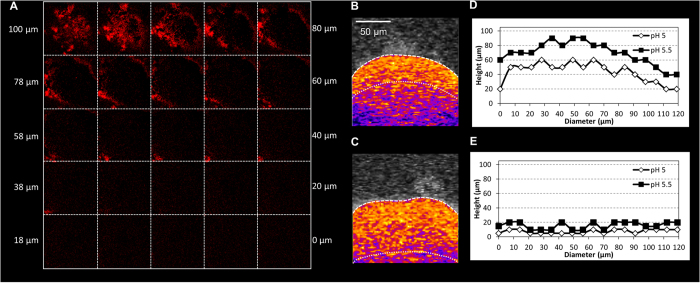
Influence of EPS matrix-degrading enzyme on the pH distribution within the microcolony . (**A**) A sequential montage of cross-sectional images of degraded EPS-matrix from top to bottom areas of the microcolony; the images of degraded EPS-matrix were obtained by determining the differences between the images before and after dextranase treatment via the fluorescence image subtraction function of ImageJ; representative orthogonal pH distribution images of (**B**) intact and (**C**) dextranase-treated microcolonies. The profile of pH values across the diameter of (**D**) intact and (**E**) dextranase-treated microcolonies. Dextranase treatment digested the upper and peripheral EPS layers, and resulted in minimal EPS degradation in the interior or bottom layers of the microcolony. Degradation of the surrounding EPS facilitates neutralization of the microcolony interior environment.

Dextranase is an EPS degrading enzyme capable of hydrolyzing α-(1→6) glucosidic linkages and branch points in both GtfB and GtfC-derived glucans^41^. These glucans have been associated with diffusion-limiting properties (albeit not clearly demonstrated yet) of cariogenic biofilms^8^,^11^,^36^. To investigate whether the EPS-matrix is connected with the creation of acidic microenvironments, we first optimized the dextranase concentration (100U) and incubation time (60 min) to degrade the surrounding EPS-matrix without disturbing the integrity of the microcolony 3D structure. Biofilm was incubated with dextranase for 60 min, followed by incubation in pH 7 buffer for another 60 min. A sequential montage of cross-sectional images from top to bottom areas of the microcolony (Fig. 6A and Fig. S1A) reveal that dextranase treatment digested the upper and peripheral EPS layers, and resulted in minimal EPS degradation in the interior or bottom layers of the microcolony, thereby maintaining its structural integrity (Fig. S1B).

The impact of the digestion of the surrounding EPS on the microcolony pH was striking. Compared to the large acidic region present in the intact microcolony (Fig. 6B), the inner acidic core (pH values below 5) was nearly abolished following dextranase treatment, while most of the microcolony interior environment was neutralized (Fig. 6C). Orthogonal pH values across the microcolony diameter showed a highly acidic region (pH 5.0–5.5) from middle to middle-upper layers of the microcolony height (Fig. 6D). In marked contrast, the majority of the dextranase-treated microcolony was neutralized, and the acidic region detected only at the bottom/deeper layers (Fig. 6E). Therefore, the data show that degradation of the EPS decreases the ability of the microcolony to withstand neutralization, demonstrating the importance of the EPS-matrix in facilitating the creation of an acidic niche.

### Simultaneous spatio-temporal analysis of pH and *atpB* gene expression level within 3D microcolony

**Figure 7:**
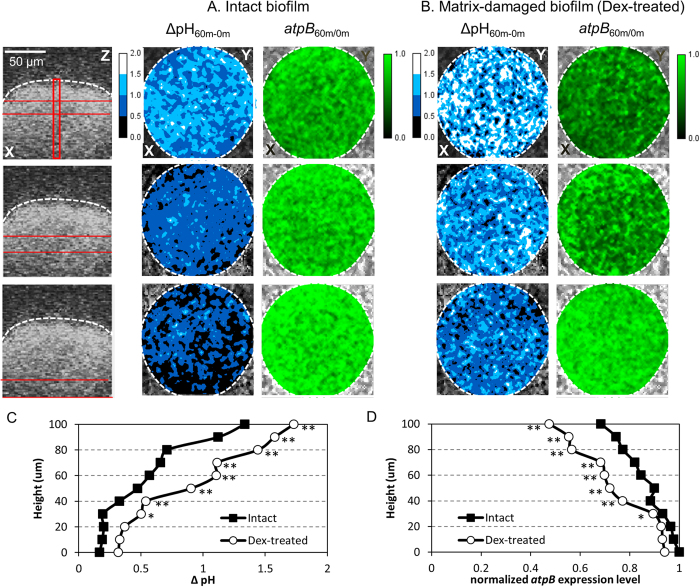
Relationship of spatial pH distribution and *atpB* gene expression within the microcolony . Cross-sectional distributions of pH and *atpB* expression levels at each selected section (red bars) within (**A**) intact and (**B**) dextranase (Dex)-treated microcolonies. We measured pH values and *atpB* expression level before (0 min) and after (60 min) exposure to neutral buffer. The results were presented as ΔpH (**C**) and fold-change *atpB* expression (**D**) (between 60 min and 0 min). The symmetric (‘mirror-image’) plots of pH (**C**) and *atpB* (**D**) from intact and Dex-treated microcolonies indicate that *S. mutans* residing within the 3D structure is actively responding to local microenvironmental changes. Asterisk (*P* < 0.05) and double asterisk (*P* < 0.01) indicate that the values for the different experimental groups are significantly different from each other.

In an acidic environment, *S. mutans* increases the production of F-ATPase (H^+^-translocating enzyme) via up-regulation of *atp* genes to pump protons out of the cells that prevent acidification of the cytoplasm, helping the bacterium to tolerate and survive the low pH conditions^42^. In *S. mutans*, the *atpB* gene encodes the beta subunit of the F-ATPase and its expression is highly induced at pH 5 while attenuated at pH 7.0^33^. Thus, to explore the localized expression of an acidic pH responsive gene within an intact microcolony, we generated a strain of *S. mutans* expressing green fluorescent protein (GFP) under the control of the *atpB* promoter (P_*atpB*_::*gfp*). We first compared *in situ atpB* gene expression and pH within an intact microcolony structure as shown in Fig. 7. We determined pH and *atpB* expression level before (0 min) and after (60 min) exposure to neutral buffer at three different layers across the center of the microcolony (see location depicted by red bars; Fig. 7A). The results were presented as ΔpH and fold-change *atpB* expression (between 60 min and 0 min).

When comparing pH values before and after exposure to neutral buffer, we found that the change in pH (ΔpH) was greater at the upper layer of the microcolony (Fig. 7A,C). Near the bottom layer, the pH changed negligibly, remaining acidic, consistent with pH mapping data (Figs 2 and 3). Concomitantly, we observed that the level of *atpB* promoter activity at the upper layer of the microcolony (more neutralized area) was significantly decreased after exposure to neutral buffer, while at the bottom layer (<40 μm) of the microcolony the *atpB* promoter remained active (Fig. 7A,D). We further confirmed the pH responsiveness of the *atpB* promoter GFP-construct. As shown in Supplementary Fig. S2, a promoter-less GFP strain showed no changes of GFP expression level across the thickness of the microcolony, indicating that GFP signal itself was unaffected by microenvironmental pH at different microcolony height. Furthermore, the levels of GFP expression from the P_*atpB*_::*gfp* construct in planktonic phase at pH 5 vs pH 7 were significantly different (Supplementary Fig. S3A). Under the acidic pH (pH 5), GFP expression was ~4 times higher when compared to the bacterial cells suspension at neutral condition (pH 7). This observation is consistent with previous studies showing up-regulation of the *atp* operon by acidic pH (~ pH 5)^33^,^34^. Finally, biofilms treated to the same experimental conditions were also tested for total *atpB* gene expression level via real-time quantitative PCR. Averaged *atpB* gene expression of whole biofilm before neutralization was ~40% higher than that after incubation at neutral pH (Supplementary Fig. S3B).

To further demonstrate the pH and matrix related changes in bacterial activity, we examined ΔpH and *atpB* gene expression within a dextranase-treated microcolony. After degradation of EPS, the changes in both pH and gene expression across the microcolony structure was significantly more pronounced as compared to the intact microcolony (Fig. 7B and open-circle plots in 7C,D). Indeed, the slope of both orthogonal ΔpH profile and *atpB* expression changes (above 40 μm) was stepper, indicating a more neutral pH and marked decrease in *atpB* (vs. intact microcolony). The symmetric (‘mirror-image’) plots of pH (Fig. 7C) and *atpB* gene expression (Fig. 7D) from both intact and matrix-degraded microcolonies indicate that *S. mutans* residing within the 3D structure is actively responding to local microenvironmental changes; and this cell-microenvironment interaction is mediated, at least in part, by the surrounding EPS-matrix.

## Discussion

The extracellular polymeric matrix is critical for the biofilm lifestyle and infectious potential of several microbial pathogens that cause many diseases in humans^1^,^6^,^10^,^11^,^21^,^43^. On tooth surface, *Streptococcus mutans* can orchestrate the assembly of caries-causing biofilms by producing EPS-matrix, while its acidogenic and aciduric properties help this microbe to create an acidic environment within plaque. The heterogeneous spatial distribution of pH within biofilms has been appreciated^36^,^44^,^45^,^46^,^47^. Recently, we reported the presence of compartmentalized acidic pH microenvironments in the interior of EPS-enmeshed microcolonies^13^. Using a surface-displayed pH sensitive fluorescent protein (pHluorins), Guo *et al.*^45^ also detected low pH values confined near the cell clusters linking our previous findings with acid accumulation within microcolonies. Here, we demonstrated that *S. mutans* EPS-matrix facilitates the generation of an acidic core within a 3D microcolony structure despite external exposure to a neutralizing buffer, which in turn activates bacterial gene expression *in situ,* mediating the biofilm microenvironment and bacterial activity locally.

The data from our study revealed an inner acidic core near the bottom layer at the center region of the microcolony, indicating that the acids, which accumulated and remained confined in this specific area were not rapidly neutralized. In contrast, the interior of the microcolony was readily neutralized when the surrounding EPS (glucans) were degraded by the topical dextranase treatment, and failed to maintain its acidic core. Thus, there is a significant resistance of neutralization that is associated with the presence of EPS. However, the precise mechanisms by which EPS contributes to maintaining an acidic microenvironment are unclear, although it appears to be a complex and multifactorial process. It has been postulated that presence of EPS limits diffusion of positively charged molecules, whereas uncharged substances such as sucrose may diffuse into biofilms^37^,^38^,^48^,^49^,^50^. Indeed, addition of sucrose resulted in rapid re-acidification of the interior of the microcolony, recreating the acidic microenvironment. The non-linear reacidification pattern across the microcolony indicated an extensive production of acids by the embedded cells as well as effective retention of acids produced, consistent with active metabolic activity of *S. mutans* within the microcolony^45^.

Furthermore, a recent study demonstrated that extracellular glucans from *S. mutans* can directly trap protons to help accumulate acids within biofilms^51^. Our data revealed that the microcolony can maintain the low pH values (despite exposure to neutral pH buffer) or be readily re-acidified as long as the structural integrity of the surrounding EPS-matrix is intact, supporting the concept that EPS help to confine extracellular acids locally. Altogether, the available evidence suggest that EPS can act a diffusion controlling barrier both by modulating the access of solutes to the interior and by trapping produced acids inside the microcolony. However, it is conceivable that some degradation of the inner layers of EPS (non-detectable using current technology) may also affect the diffusion properties. Thus, detailed studies using super-high resolution microscopy combined with 3D tracking of fluorescently-labeled solutes transport are required to further investigate the EPS-mediated diffusion/trapping mechanism. Future investigation should also examine the influences of other matrix components (such as fructans and eDNA) and the presence of other bacteria in mixed-species biofilm models.

In parallel, the generation of acidic pH niches has a local impact upon bacterial activity within the intact 3D microcolony. Using a GFP-reporter driven by the *atpB* promoter together with pH mapping analysis, we examined *in situ* how the bacterial activity (via gene expression) coordinates with local changes of the microcolony microenvironment. Our results demonstrate that *S. mutans* can rapidly adapt itself spatiotemporally within a dynamically changing 3D milieu via inherent proton pumping function of the F-ATPase. In turn, it can help this pathogen survive the acidic stress caused by localized acid accumulation due to the presence of the EPS-matrix. While this study focused on changes of gene expression in the context of pH, it remains to be determined how *S. mutans* responds to other stresses and constituents of the oral cavity (e.g. other bacteria and their byproducts), which may affect the overall capacity of this oral pathogen to colonize and cause disease. Nevertheless, the possibility to assess the microenvironment and gene expression simultaneously opens new opportunities to investigate the intricate bacteria-niche interactions within intact biofilms.

In conclusion, our study provides new insights on co-dependency and interconnected relationship between the biofilm matrix, local microenvironment and bacterial activity *in situ* at a single microcolony level. At the same time, we demonstrate a potentially useful and powerful analytical tool for biofilm research. The data also emphasize the critical role of *S. mutans*-derived EPS in modulating both the biofilm matrix properties and the pH microenvironment, which can promote local acidification and creation of a favorable niche for the growth of an acidic microbiota that may eventually lead to dental caries. Because EPS is also linked with viscoelastic properties and attachment strength of biofilms^9^, targeting the mechano-chemical properties of the matrix and the derived microenvironment may lead to exciting new anti-plaque and anti-caries therapeutic strategies.

## Materials and Methods

### Bacterial strains

*Streptococcus mutans* UA159 (ATCC 700610), a virulent cariogenic pathogen and well-characterized exopolysaccharides (EPS)-matrix producer and acidogenic/aciduric strain, was used to generate biofilms using saliva-coated hydroxyapatite (sHA) model^13^,^52^. Furthermore, a promoter-less green fluorescent protein (GFP)-expressing strain and an *atpB* promoter-GFP construct (P_*atpB*_::*gfp*) of *S. mutans* UA159 were used for *in situ* gene expression. The cultures were stored at −80 °C in tryptic soy broth containing 20% glycerol.

### Construction of *S. mutans* expressing green fluorescent protein

The GFP-expressing variant of *S. mutans* UA159 was constructed using the pUG plasmid containing a promoter-less *gfp* gene (from the jelly fish *Aequorea victoria,* a gift from Z. T. Wen, Louisiana State University). The 186 bp region immediately upstream the *atpB* gene comprising the promoter region was amplified using the primer pair atpB_GFP_BamHI 5′CCAACTCTAATTCCGGATCCTCTTATTATG 3′and atpB_GFP_EcoRI 5′GATTTTAGCACTTGGAATTCCTGTTTTTAGG 3′. Underlined sequences indicate restriction enzyme sites introduced for cloning purposes. The PCR product and the pUG plasmid were digested with BamHI and EcoRI, subsequently ligated and introduced into *Escherichia coli* DH10B cells for replication. Positive transformants were grown in agar plates containing 100 μg ml^−1^ ampicillin and confirmed by PCR and sequencing analyses. Once the pUG::P*_atpB_* plasmid was constructed, we used the integration vector pMC340B^53^ to introduce the promoterless *gfp* and the P*_atpB_*::*gfp* products into *S. mutans*. For this, the *gfp* and P*_atpB_*::*gfp* regions were amplified from pUG using primers pUG XhoI F 5′ GACGTTGTAACTCGAGGGCCAGTG 3′ and pUC18ermcassette-2 5′ GGCTCGTATGTTTGTGTGG 3′. These products were digested with *SphI* and *XhoI*, subsequently ligated to the pMC340B plasmid and then transformed into *E. coli* DH10B cells. The pMC340B::*gfp* and pMC340B::P*_atpB_-gfp* constructs were selected on plates containing 100 μg ml^−1^ kanamycin and confirmed by PCR and sequence analyses. Next, the pMC340 was used to transform *S. mutans* UA159. Positive transformants were selected on BHI plates containing kanamycin 1 mg ml^−1^. The integration of promoterless *gfp* and P*_atpB_*::*gfp* at the *mtlA* locus was confirmed by PCR, sequence and GFP expression analyses. Because promoterless *gfp* and P*_atpB_*::*gfp* were stably integrated in the chromosome, downstream experiments were conducted in the absence of antibiotic pressure.

### Biofilm preparation

Biofilms were formed on saliva-coated hydroxyapatite (sHA) discs (surface area, 2.7  ± 0.2 cm^2^; Clarkson Chromatography Products, Inc., South Williamsport, PA, USA) as detailed previously^52^. sHA discs were vertically suspended in 24-well plates using a custom-made wire disc holder. Each disc was inoculated with ~2 × 10^5^ CFU of *S. mutans* ml^−1^ in ultrafiltered (10 kDa molecular mass cut-off; Millipore, Billerica, MA) yeast tryptone extract broth (UFTYE; 2.5% tryptone and 1.5% yeast extract at pH 7.0) containing 1% (30 mM) sucrose at 37 °C and 5% CO_2_. The 3D biofilm architecture and the spatial organization of EPS were assessed using confocal imaging^13^,^52^. Briefly, Alexa Fluor 647 dextran conjugate (Molecular Probes Inc., Eugene, OR) was added to the culture medium during the formation of *S. mutans* biofilms. The fluorescently labelled dextran serves as a primer for Gtfs, and is directly incorporated into glucans during EPS matrix synthesis over the course of biofilm development as demonstrated in previous studies^13^,^52^. During the first 19 h, the cells were grown undisturbed to allow initial biofilm formation; the culture medium was then replaced twice daily at 8 am and 6 pm until the end of the experiment period (42 h). The pH of the culture medium was recorded daily at each medium change.

### Non-invasive 3D *in situ* pH measurement via multi-photon confocal microscopy

To stain EPS and measure the *in situ* biofilm pH, we used fluorescent pH-indicator Lysosensor yellow/blue (Molecular Probes Inc., Eugene, OR) labeling method as detailed previously^13^. Briefly, biofilms were incubated with lysosensor yellow/blue dextran conjugate, and the pH values within intact biofilms were measured based on fluorescence intensity ratios of the dual-wavelength fluorophore. The fluorophore exhibits a dual-emission spectral peak (fluorescence emission maxima 452 nm and 521 nm), and the ratio between the fluorescence intensity of these two spectral peak is pH-dependent within biofilms^13^. The fluorescence intensity of both emission wavelengths and the ratio of fluorescent intensity (I450/I520) within each biofilm image were measured using Image J 1.44 and its calculation tools (http://www.uhnresearch.ca/facilities/wcif/PDF/ImageJ_Manual.pdf). Titration curves of ratios versus pH (ranging from 4.0 to 7.0) were determined as described previously^13^. The confocal images of lysosensor-incorporated biofilms were acquired before and after incubation in Na_2_HPO_4_-citric acid buffer at pH 7.0 using a multi-photon laser scanning microscope (SP5, Leica Microsystems, Buffalo Grove, IL, USA) equipped with a 20X (1.0 numerical aperture) water immersion lens. The biofilms were excited at 700 nm, and emission was detected in two channels: one at 450 nm (using non-descanned detector NDD1; −495 nm) and the other at 521 nm (using non-descanned detector NDD2; 495–560 nm). The images were acquired after 10, 30, and 60 min of incubation in the neutral buffer to evaluate the spatio-temporal distribution of pH throughout the intact 3D biofilm architecture. For pH measurement, the ratios of fluorescence intensity of selected areas within each biofilm image were converted to pH values using the titration curves and Image J. For visualization of *in situ* pH distribution within the 3D biofilm architecture, the fluorescence intensity ratios (corresponding to the pH values between 7.0 and 4.0) of all confocal images were reconstructed using Image J, then Amira. The fluorescence intensity was converted into grayscale using the Amira tool-box to correlate with the pH range from 7.0 (white) to 4.0 (black)^13^. For enhanced contrast, we transformed the grayscale into different range of colors by applying ImageJ’s lookup table (LUT)^39^; Red Hot LUT was used for the pH range within microcolony (Figs 2, 3, 4); Fire LUT was used for the detection of acidic core within microcolony (Figs 5 and 6); modified Cyan Hot LUT was used for the visualization of ΔpH within microcolony (Fig. 7). Unlabeled biofilms were also imaged to determine whether the autofluorescence of each biological component of the biofilm (i.e. bacterial cells and glucans) would interfere with pH quantification at the wavelengths and laser intensity used in our study; there was no interference with the measurements based on our image analysis.

### Biofilm re-acidification and treatment with EPS-degrading enzyme

For the biofilm re-acidification experiment, the biofilm formed on sHA was incubated in the neutral pH buffer for 60 min and then supplemented with 1% (30 mM) sucrose for another 60 min to promote acid production by *S. mutans* within microcolonies. The *in situ* pH was measured and visualized as described above. For EPS-matrix degradation experiment, biofilms were treated with an EPS-degrading enzyme (dextranase; EC 3.2.1.11; Sigma-Aldrich, St. Louis, MO, USA) that is capable of hydrolyzing α-(1→6) glucosidic linkages and branch points in both GtfB and GtfC-derived glucans^41^. Briefly, biofilms were incubated with 100U of dextranase in Na_2_HPO_4_-citric acid buffer at pH 5 at 37 °C for 60 min. This experimental condition was used based on optimal enzyme amount to degrade the surrounding EPS-matrix without affecting bacterial viability or disrupting the 3D architecture of the biofilms as determined via viable cell counting (data not shown) and confocal microscopy (Supplementary Fig. S1). After dextranase treatment, biofilms were washed three times with the Na_2_HPO_4_-citric acid buffer at pH 5.0 to remove residual dextranase, followed by incubation in the neutral pH buffer for 60 min. The images were acquired after 10, 30, and 60 min of incubation, and the pH throughout the 3D biofilm architecture analyzed as described before. Differences of EPS-matrix before and after dextranase treatment were determined via the fluorescence image subtraction function of ImageJ.

### Gene expression levels within biofilms via *atpB*::*gfp* reporter

For measurement of *atpB* promoter activity of bacterial cells within the microcolony, the biofilms were sequentially scanned using the 488 nm Argon laser to minimize the crosstalk between GFP and Lysosensor, and the fluorescence emitted was collected with the internal spectral detectors (515–545 nm). For visualization of gene expression level, ImageJ’s Green LUT was applied (Fig. 7).

### Statistical Analyses

Four independent biofilm experiments were performed, and 10 image stacks (512×512 pixel for quantification in tagged image file format) were collected for each experiment. The data were analyzed by pairwise comparisons of multiple groups with regression models using ranked values. Kruskal-Wallis tests, which are non-parametric and based on ranks, were used for two-group comparisons. The significance levels were set at 5 and 1%.

## Additional Information

**How to cite this article**: Hwang, G. *et al.* Simultaneous spatiotemporal mapping of *in situ* pH and bacterial activity within an intact 3D microcolony structure. *Sci. Rep.* **6**, 32841; doi: 10.1038/srep32841 (2016).

## Supplementary Material

Supplementary Information
