# Supplementary material for: Simultaneous spatiotemporal mapping of in situ pH and bacterial activity within an intact 3D microcolony structure
Source: Sci Rep. 2016 Sep 8;6:32841. doi: 10.1038/srep32841 (PMC5015094; doi:10.1038/srep32841)
Supplement: Supplementary Information [file srep32841-s1.pdf]

Supplementary information:

Simultaneous spatiotemporal mapping of *in situ* pH and bacterial activity within an intact 3D microcolony structure

Geelsu Hwang[1*], Yuan Liu[1], Dongyeop Kim[1], Victor Sun[1], Alejandro Aviles-Reyes[2], Jessica K. Kajfasz[2], Jose A. Lemos[2], Hyun Koo[1*]

[1]*Biofilm Research Labs, Levy Center for Oral Health, Department of Orthodontics and Divisions of Pediatric Dentistry & Community Oral Health, School of Dental Medicine, University of Pennsylvania, Philadelphia, PA, USA*

[2]*Department of Oral Biology, University of Florida College of Dentistry, Gainesville, FL, USA*

* Corresponding authors

Geelsu Hwang, 240 South 40th Street, Levy Bldg. Rm 418, Philadelphia, PA 19104-6030; Tel: (215) 898-1571; E-mail: geelsuh@upenn.edu; Hyun (Michel) Koo, 240 South 40th Street, Levy Bldg. Rm 417, Philadelphia, PA 19104-6030; Tel: (215) 898-8993; E-mail: koohy@upenn.edu

Prediction of diffusive penetration time of solutes into biofilm

In biofilms, diffusion (random molecular motion) is a primary driving force for transporting solutes into a bacterial cluster (microcolony) [1]. To estimate how fast the solutes from the buffer (citric acid-Na_2HPO_4) reach to the core of microcolony, we employed a simple diffusion model of biofilm [1]. As we observed from confocal images (Fig. 1), the biofilms consist of densely packed mushroom-shaped bacterial clusters. Although the microcolonies are not uniformly distributed across the surface, we assumed the biofilms are composed of same height microcolonies. In this study, we calculated diffusive penetration time of solute into three different microcolony thicknesses, such as 100, 60, and 20 μm. Given the geometry of the system, the time required for a solute to attain at the base of biofilm is given by

$$t_{90} = 1.03\frac{L^2}{D_p} \qquad (1)$$

where L is the biofilm thickness, and D_p is the effective diffusive permeability of the pure EPS phase [2]. D_p is estimated from the diffusion coefficient in pure water (D_{aq}), and its relationship for a small solute is described by [2]

$$D_p/D_{aq} = 0.025 \qquad (2)$$

Aqueous diffusion coefficients, penetration time depending on the microcolony size used in this study are summarized in Table 1.

Table S1. Diffusion coefficients used in this study, and estimated penetration times depending on the microcolony thickness.

Solute	10^{-6} D_{aq} [cm^2/s]	Thickness of microcolony [µm]	t_{90} [t]
Citric acid	6.57[3]	100	627
		60	226
		30	56
HPO_4^{2-}	7.59[4]	100	543
		60	195
		30	49

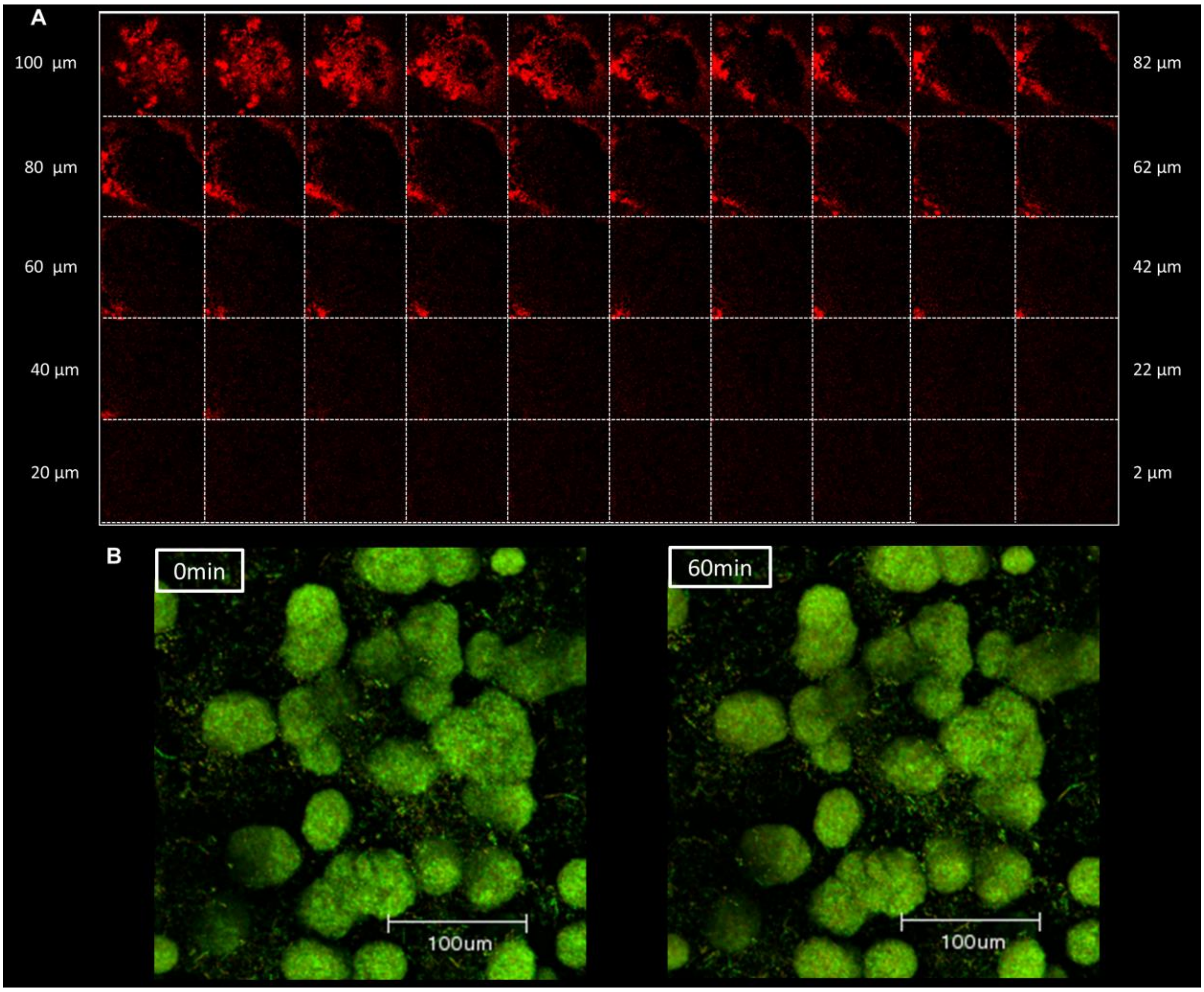

Figure S1. Influence of EPS matrix-degrading enzyme on the biofilm. (A) A sequential montage of cross-sectional images of degraded EPS-matrix from top to bottom of microcolony. The images of degraded EPS-matrix were obtained by determining the differences between the images before and after dextranase treatment via the fluorescence image subtraction function of ImageJ. (B) 3D images of bacterial cells before (0 min) and after (60 min) application of dextranase, indicating that the overall 3D structure of the microcolony is not affected by the digestion of the surrounding EPS-matrix.

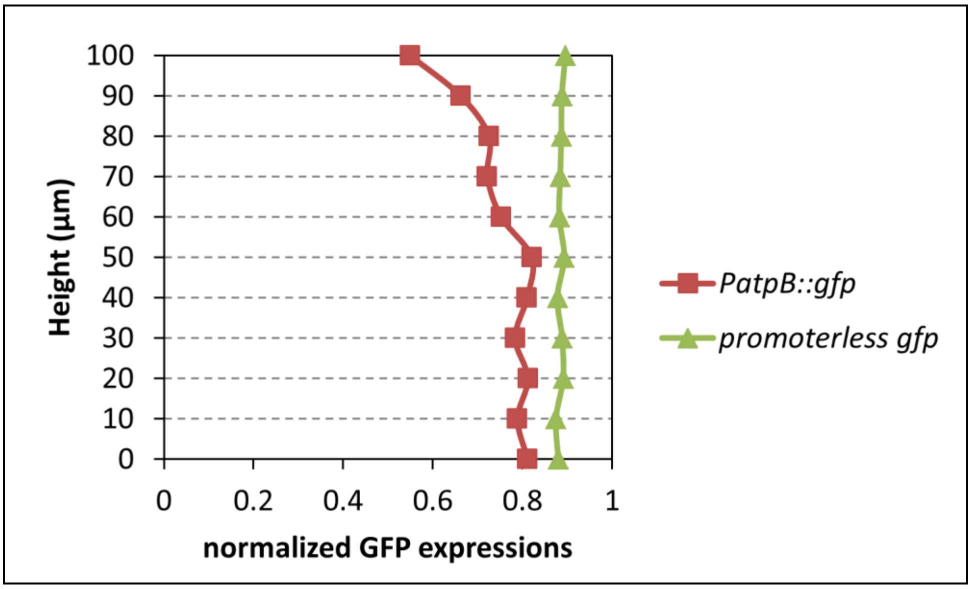

Figure S2. GFP expression profiles at the center of microcolony. Green line indicates the profile of a promoter-less GFP *S. mutans*, while the red line depicts the profile of P*atpB::gfp* strain across the thickness (height) of the microcolony.

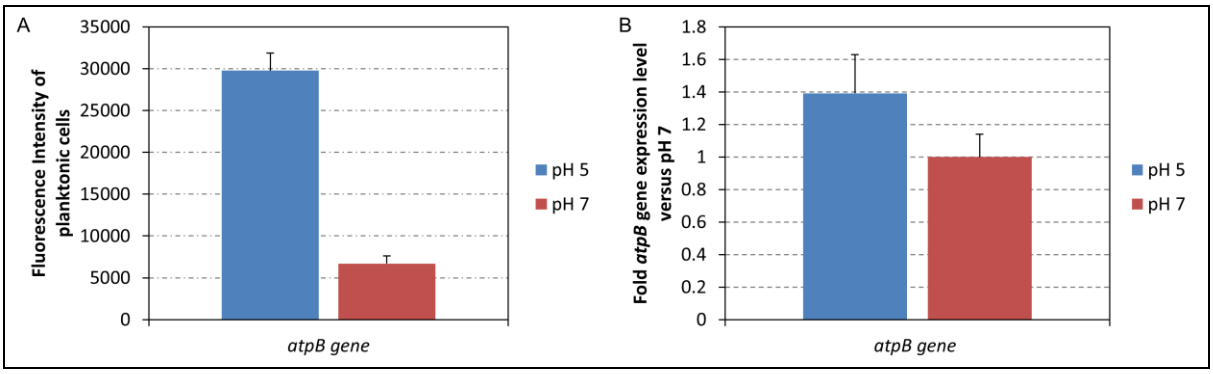

Figure S3. *atpB* gene expression level of *S. mutans* under acidic (pH 5) versus neutral conditions (pH 7). (A) Fluorescence intensity of *S. mutans* planktonic cells. (B) Fold change of *atpB* gene expression level of *S. mutans* biofilms by qRT-PCR.

References

1. Stewart, P. S. Diffusion in biofilms. *J. Bacteriol.* **185**, 1485-1491 (2003).

2. Stewart, P. S. A review of experimental measurements of effective diffusive permeabilities and effective diffusion coefficients in biofilms. *Biotechnol. Bioeng.* **59**, 261-272 (1998).

3. Müller, G. & Stokes, R. The mobility of the undissociated citric acid molecule in aqueous solution. *T. Faraday Soc.* **53**, 642-645 (1957).

4. Southard, M. Z., Dias, L. J., Himmelstein, K. J. & Stella, V. J. Experimental determinations of diffusion coefficients in dilute aqueous solution using the method of hydrodynamic stability. *Pharm. Res.* **8**, 1489-1494 (1991).
